# The Role of PPAR*γ* in Hepatocellular Carcinoma

**DOI:** 10.1155/2008/209520

**Published:** 2008-05-26

**Authors:** Ivan Borbath, Yves Horsmans

**Affiliations:** Gastroenterology Unit, Cliniques universitaires Saint-Luc, Université catholique de Louvain, Avenue Hippocrate 10, 1200 Bruxelles, Belgium

## Abstract

Hepatocellular carcinoma (HCC) is the third leading cause of cancer-related death worldwide. This cancer develops mainly in cirrhotic patients. The cirrhotic liver is considered to be a preneoplastic organ, suggesting the rationale for cancer prevention. PPAR*γ* is a nuclear transcription factor whose activation leads to interaction in the metabolism of lipids, insulin sensitization of peripheral cells, anti-inflammatory action. It can also induce differentiation and inhibits proliferation of cancer cells. Until now, data using PPAR*γ* ligands in HCC have demonstrated mainly in in vitro models that its activation could be due to an antiproliferative effect. PPAR*γ* ligand administration has also been associated with a diminution of liver fibrosis in animal models, and potentially also on tumoral cell death. Soma data show that the favorable effect of natural and synthetized PPAR*γ* agonists could also be independent of PPAR*γ* activation. Furthermore, in some situations, PPAR*γ* antagonists have also an anticancer effect. Therefore, we can conclude that the link between activation of the PPAR*γ* pathway and an anticancer activity is suggested but until now not firmly established in HCC.

## 1. INTRODUCTION

Hepatocellular carcinoma (HCC) is the third leading cause of cancer-related death worldwide [1]. Its prognosis is poor, with up to 40% of patients diagnosed at an advanced stage, when only palliative treatments can be offered. Most patients that develop HCC have cirrhosis, considered as a preneoplastic organ. Strategies to prevent the development of HCC have been implemented, by preventing the appearance of cirrhosis (e.g., HBV vaccination and treatment) [2] or the reappearance of HCC in cirrhotic patients (e.g., interferon in HCV-related cirrhotic patients) [3].

PPAR*γ* is a nuclear transcription factor, member of the nuclear hormone receptor superfamily, being activated by binding its ligand, then heterodimerizing with the Retinoid X receptor, to finally bind with specific response elements in the nucleus, called peroxisome proliferating response elements. Activation of PPAR*γ* by agonists such as thiazolidinediones (TZDs) has been shown to have anticancer effect in vitro and in vivo in many cancer types [4]. However, as emphasized by articles in this special issue of PPAR research, in some situations, like in colon cancer models, PPAR*γ* agonists can have tumor promoting effects [5].

Effect of PPAR*γ* agonists on HCC has been studied in the last years both in cell cultures and in animal models. Here, we summarize the main findings of these works and attempt to define the role of PPAR*γ* and its modulation in HCC.

## 2. EXPRESSION OF PPAR*γ* IN RODENT AND HUMAN LIVER

PPAR*γ* RNA and protein expression has been demonstrated in vitro in several cell lines, including Hep3B, HepG2, Huh7, and others [6–11]. Its expression was generally higher than positive control used but variable among cell lines, with mRNA levels not necessarily correlating protein expression.

In human HCC, reports from 3 papers show conflicting results: in the first paper, PPAR*γ* protein expression was assessed by western-blot (WB) in 5 cirrhotic patients and showed no difference between HCC and nontumoral cirrhotic liver [12]. The second paper showed a constant overexpression of PPAR*γ* protein in 20 HCCs and no expression in surrounding liver, assessed by immunohistochemistry (IHC). In this paper, no information was given regarding the presence of cirrhosis [13]. Finally, a recent report, analyzing mRNA and protein expression (by WB) in 20 patients, with cirrhosis (*n* = 12) and chronic hepatitis (*n* = 8), showed a statistically significant decrease in PPAR*γ* expression in HCC compared to adjacent liver [14]. Furthermore, PPAR*γ* mRNA was less expressed in poorly differentiated HCC compared to well-differentiated ones. The latter [2] results, using 2 different methods (IHC versus WB) in different populations, can therefore not be compared, and certainly other analyses will be needed before we can draw any conclusions on PPAR*γ* expression in HCC and cirrhosis compared to normal liver, where it is known to be low [15].

## 3. EFFECT OF PPAR*γ* AGONISTS ON CELL PROLIFERATION

The effect of the natural PPAR*γ* agonist 15-deoxy-Δ12,14-prostaglandin J2, and the synthetic agonists thiazolidinediones has been tested in many human HCC cell cultures [6, 7, 9, 11, 12, 14, 16, 17]. Overall, cell growth was inhibited at variable concentrations. Troglitazone, the most studied TZD, was shown to inhibit cell growth already at 5–10 *μ*Mol concentrations, although some contradictory reports have been published [10]. Pioglitazone and ciglitazone, the other main TZDs tested, were less active, with concentrations from 50 to 100 *μ*Mol needed to have significant growth inhibitory effect. The antiproliferative effect was shown to be due to a cell cycle arrest, with cells accumulating at the G1 phase. Koga et al. have performed the most thorough mechanistic investigations in the field [7, 12]. Using Troglitazone at a dose of 50 *μ*Mol, they could show on several cell lines that the inhibitory effect on cell cycle was linked to an increase in the mRNA and protein expression of the Cyclin-dependent kinase inhibitor (Cki) p21^waf1/cip1^ and also an increase in protein expression only of p27^kip1^, another CKi. These proteins were shown to bind to the Cyclin-dependent kinase CDK2, thus inhibiting its activation. Because the intracellular concentration of p27^kip1^ is tightly regulated by its degradation through the ubiquitin-proteasome pathway, the authors have analyzed and could show that indeed, troglitazone had an inhibitory effect on p27^kip1^ degradation via inhibition of Skp2, an F-box protein of ubiquitin-ligase complex. These results were reproduced by others [11, 14, 18].

In vivo, four papers showed an inhibition of the development of preneoplastic and neoplastic liver nodules by PPAR*γ* agonists. Kawaguchi et al. [19], using pioglitazone in male Wistar rats fed with a choline-deficient L-amino acid defined diet, which is an animal model of nonalcoholic steatohepatitis (NASH) model, showed that pioglitazone, at a concentration of 0.01% wt/wt, inhibited the formation of GSTp positive foci by a factor 2. GSTp, the placental form of Glutathione S-transferase, is an early marker of malignant transformation, widely used in carcinogenic models [20]. Guo et al. [21], using three TZDs, namely, troglitazone, rosiglitazone, and pioglitazone, in male Wistar rats showed that all could suppress the formation of GSTp foci that were induced by the strong mutagen diethylnitrosamine (DEN). In a longer experiment aiming at tumor formation, they found that pioglitazone at a 200 ppm concentration, equivalent to 0.05% wt/wt, prevented the appearance of macroscopic tumors. However, in this paper, no histologic proof of hepatocellular carcinoma was demonstrated, and the quantitation of tumors is subject to criticism, with a “stereomicroscopical” method used that is not described. We recently published our results [22], using pioglitazone at a dose of 0.01% wt/wt, in a carcinogenic 2-stage rat model, using DEN and acetylaminofluorene. We could show that given early in the carcinogenic process, pioglitazone inhibited the formation of preneoplastic foci to 50% of the control group. This was accompanied by a significant decrease of proliferation in the transformed foci, and an overexpression of the CKi p27^kip1^ in the pioglitazone-treated group compared to control, thus reproducing some of the results obtained in vitro. Finally, Yu et al. published their results on the effect of troglitazone, in vitro and in vivo in a nude mice model, injected subcutaneously with the human Huh7 HCC cell line [14]. They could show quite convincingly that troglitazone, at the 200 ppm concentration, either inhibited the appearance of HCC, or decreased the growth of existing ones. The authors demonstrated that troglitazone induced a decrease in proliferation, assessed by PCNA, an increase in p27^kip1^ and a decrease of COX-2 expression in tumors compared to controls.

It is important to mention that none of these studies, using TZDs, has clearly demonstrated the antiproliferative effect of these drugs to be due to activation of PPAR*γ* pathway. In other cancer types, authors have demonstrated that troglitazone and ciglitazone could inhibit cell proliferation in PPAR*γ* ^−/−^ cell lines, by inhibition of translation initiation [23].

## 4. ROLE OF PPAR*γ* IN THE PROGRESSION OF LIVER FIBROSIS

An important paper published in 2002 by Galli et al. [24] demonstrated that, given early, TZDs could significantly reduce the development of liver fibrosis induced in rats, either by chronic administration of dimethylnitrosamine, carbon tetrachloride, or bile duct ligation. The authors could show that this antifibrotic effect was driven through a PPAR*γ*-dependant decreased activation of hepatic stellate cells. These early results showing the importance of PPAR*γ* activation in the pathogenesis of fibrosis were confirmed by others [25, 26]. Though, we could show that the protective effect was very dependant on the rodent type [27] and the severity of fibrosis at the time of initiation of treatment [28]. These data are thus to be interpreted with caution before going to clinical application. In humans, one randomised double-blind placebo-controlled study assessed the effect of pioglitazone versus placebo in patients with NASH [29]. One of the aims of the study was to assess fibrosis on liver biopsy before and after 6 months of a calorie-restricted diet with or without pioglitazone. The study failed to show differences in terms of fibrosis regression, assessed by histology, between groups (*P* = 0.08), although patients in the pioglitazone group showed significantly less fibrosis after the 6-month regimen compared to before treatment (*P* = 0.002).

## 5. PPAR*γ* MODULATION IN ASSOCIATION WITH CELL DEATH

The role of PPAR*γ* in apoptosis and anoikis is less clear. Several in vitro works report induction of apoptosis in HCC cell lines, constantly by troglitazone and not by others TZDs [9, 14, 30–32]. The main apoptotic mechanisms remain poorly understood, although some publications report an effect of troglitazone on the bax/bcl-2 balance [32], suggesting a role for the mitochondrial pathway. In another work, authors have analyzed the effect of the natural PPAR*γ* agonist 15-deoxy-Δ12,14-prostaglandin J2 (15D-JG2) on apoptosis of SK-Hep1 and hepG2 cells. They indeed showed that given at a dose of 50 *μ*Mol, 15D-JG2 could induce apoptosis by caspase 3 activation. Using PPAR*γ* antisense oligodeoxynucleotides, they demonstrated that apoptosis was induced independently of PPAR*γ* expression. They also showed that 15d-PGJ2 inhibited NF-kB activation induced by TNF*α* when PPAR*γ* was normally expressed or downregulated.

## 6. IS THERE A ROLE FOR PPAR*γ* INHIBITION IN CANCER?

Although, as discussed earlier in the text, the vast majority of available data deals with PPAR*γ* agonists, some authors analyzed the effect of PPAR*γ* inhibitors on cancer cell growth. One publication reported the effect of PPAR*γ* inhibitors on cell adhesion and anoikis [13]. On the contrary to the paper by Yu et al. [14], authors found overexpression of PPAR*γ* in human HCC. They could show that these PPAR*γ* inhibitors increased loss of adherence in vitrofollowed by caspase-dependent apoptosis, a finding reproduced using PPAR*γ* siRNAs, reflecting the specificity of the PPAR*γ*-dependent activation driving cell death. They also showed that inhibitors were at least 5-fold more potent in reducing cell number than troglitazone and rosiglitazone. Another study evaluated the effect of PPAR*γ* agonists and antagonists on cell growth, migration, and invasion in four different HCC cell lines [33]. Authors could show that antagonists inhibited cell growth and migration more efficiently than PPAR agonists. They suggest that this effect could be due to Vimentin cleavage, thus interacting negatively with the cellular cytoskeleton.

## 7. CONCLUSION

Several in vitro and in vivo papers suggest a role of the PPAR*γ* pathway in the prevention and treatment of HCC. PPAR*γ* mRNA and protein expression are constantly found in cell lines. In normal liver, it is known to be expressed at low levels, ten times less than in adipose tissue. We do not know its actual level of expression, neither in cirrhotic nontumoral liver, nor in HCC, with conflicting results reported. Thiazolidinediones, which are active and specific PPAR*γ* agonists, have shown antitumoral activity in cell lines and in animal models. This anticancer activity is due to inhibition of cell proliferation, by interfering with important cell cycle cyclins and cyclin-dependent kinase inhibitors, such as p27^kip1^, but also by a proapoptotic action. The link between activation of the PPAR*γ* pathway and the anticancer activity of TZDs is, however, not established. Finally, PPAR*γ* antagonists have shown antitumor activity, by different mechanisms, mainly involving loss of cell adherence, migration, and invasion.
